# Effects of a daily, home-based, 5-minute eccentric exercise program on physical fitness, body composition, and health in sedentary individuals

**DOI:** 10.1007/s00421-025-05757-7

**Published:** 2025-03-25

**Authors:** Benjamin J. C. Kirk, Georgios Mavropalias, Anthony J. Blazevich, Jodie L. Cochrane-Wilkie, Aus Molan, Kazunori Nosaka

**Affiliations:** 1 School of Medical and Health Sciences, Edith Cowan University, Joondalup, WA Australia; 2 Physical Activity, Sport and Exercise Research Theme, Faculty of Health, Southern Cross University, Lismore, NSW Australia; 3 PathWest Laboratory Medicine, Nedlands, WA Australia; 4 Exercise Medicine Research Institute, Edith Cowan University, Joondalup, WA Australia

**Keywords:** Isometric mid-thigh pull, Push-up, Sit-up, Sit-and-reach, 3-Min step test, 36-Item short form survey (SF-36)

## Abstract

**Purpose:**

This study examined the effects of a 4-week home-based bodyweight eccentric exercise program, requiring just 5 min daily, on physical fitness, body composition, and both physical and mental health in sedentary individuals.

**Methods:**

Twenty-two sedentary but healthy individuals (4 men, 18 women; 32–69 years) completed a two-week control period followed by a 4-week intervention. The intervention involved daily exercises consisting of 10 repetitions each of chair squats, chair reclines, wall push-ups, and heel drops, including progressions if necessary. Assessments included isometric mid-thigh pull (IMTP), handgrip (HG) strength, push-up and sit-up endurance, sit-and-reach (S&R) flexibility, 3-min step test (3ST), squat jump (SJ), countermovement jump (CMJ), body composition (via dual-energy X-ray absorptiometry), physical health markers (blood based), and mental wellbeing (SF-36 survey and subjective vitality scale [SVS]). Measurements were taken before (PRE-1), after the control period (PRE-2), and after the 4-week training period (POST).

**Results:**

Adherence to the program was 91±12% (18–28 sessions over 28 days). Intraclass correlation coefficients presented moderate-to-excellent reliability within the control period. No significant changes were observed in body composition, resting heart rate, blood pressure, HG, SJ, CMJ, or blood markers after training. However, significant improvements (p < 0.05) were noted in IMTP (13.0±18.5%), push-up (66.1±86.5%), sit-up (51.1±78.7%), S&R (9.1±20.0%), and 3ST heart rate (4.8±7.1% decrease). Mental health scores improved significantly (SF-36 by 16±29% and SVS by 20±3%, p < 0.05).

**Conclusion:**

The 5-minute daily eccentric exercise routine over 4 weeks significantly improved physical fitness and mental health in sedentary individuals, suggesting even a small dose of daily exercise can be beneficial.

**Supplementary Information:**

The online version contains supplementary material available at 10.1007/s00421-025-05757-7.

## Introduction

Physical inactivity is a leading cause of morbidity and mortality, contributing to the prevalence of chronic diseases, and is estimated to cause 9% of premature deaths globally, which amounts to more than 5.3 million deaths annually (Lee et al. 2012). This highlights the urgent need for effective strategies to promote regular physical activity across diverse populations. The World Health Assembly in 2018 sanctioned the Global Action Plan on Physical Activity (GAPPA) for the period 2018–2030 and adopted a new voluntary global target to reduce global levels of physical inactivity in adults and adolescents by 15% by 2030 (World Health Organization 2019). Multiple professional organizations have complemented these global initiatives by issuing their own guidelines for recommended physical activity levels for healthy youth (Faigenbaum et al. 2009), adults (Garber et al. 2011), older adults (Chodzko-Zajko et al. 2009), and individuals with chronic diseases (Kanaley et al. 2022; Selig et al. 2010). Overall, these guidelines recommend that adults aged 18–64 accumulate at least 150 minutes of moderate-intensity aerobic physical activity or 75 minutes of vigorous intensity aerobic physical activity each week, or an equivalent combination of both moderate and vigorous activities, and 1–3 sets of 8–15 repetitions of 8–10 resistance exercises on 2 or 3 days per week (Garber et al. 2011; Bull et al. 2020).

Despite these recommendations, a significant portion of the population fails to meet the suggested guidelines for both general activity (Australian Institute of Health and Welfare 2023; Bennie et al. 2015) and resistance exercise participation (Nuzzo 2023). For example, a review of adult physical activity in the United States revealed that only 30% of people met the aerobic exercise guidelines, 10% met the muscle strengthening activity recommendations, and 40% failed to meet any of the guidelines (Bennie et al. 2019). Similarly in Australia, a review of adult physical activity revealed that while 63% of people met the aerobic guidelines, only 29% met the muscle strengthening activity criteria and just 19% met all the guidelines (Berlingeri et al. 2017). Moreover, approximately 50% of individuals revert to physical inactivity or a less active state within the first few months after initiating an exercise regimen (Dishman and Buckworth 2013; Marcus et al. 2000). Perceived lack of time is one of the most frequently cited barriers to exercise participation (Bowles et al. 2002; Hoare et al. 2017) and is also a common reason why individuals do not adhere to exercise prescriptions and why some individuals discontinue exercise (Gettman et al. 1983; Van Roie et al. 2015). To address this challenge, it is essential to explore exercise modalities that are time efficient yet effective in improving health outcomes.

Resistance training could offer a promising solution in this context. Unlike traditional aerobic exercises that often require longer durations, resistance exercises, including minimal-dose strategies such as single-set resistance exercise or eccentric exercise approaches, can provide significant health benefits with shorter time commitments (Sato et al. 2022; Yoshida et al. 2022; Katsura et al. 2019; Nuzzo et al. 2024). Such a minimal-dose strategy, focusing on simple yet effective movements, may be particularly suitable for sedentary individuals with limited time, and could provide a gateway to regular exercise participation, helping individuals overcome barriers associated with perceived lack of time.

Eccentric resistance exercise has been recognized as a potent method for enhancing muscle size and strength (Roig et al. 2009; Schoenfeld et al. 2017; Douglas et al. 2017), and improving joint flexibility (Diong et al. 2022; Kay et al. 2023). Moreover, studies suggest that when eccentric and concentric-focused resistance exercise are completed separately with equal absolute workloads, cardiovascular stress and perceived effort are lower during eccentric exercise (Hollander et al. 2003; Miller et al. 2009; Vallejo et al. 2006). This lower perceived effort may make eccentric exercise more appealing and sustainable, particularly for individuals who are new to exercise or have struggled to maintain consistent physical activity. The reduced physical strain could help lower barriers exercise adherence, making it easier for people to start and maintain a regular exercise routine, thereby promoting long-term engagement in physical activity.

Previous research supports these benefits, showing that even brief, time-efficient eccentric exercise interventions can lead to substantial physical and health improvements. For example, Sato et al. (2022) demonstrated that a single 3-second maximal eccentric contraction of the elbow flexors, performed five days a week for four weeks, significantly enhanced muscle strength. Similarly, Yoshida et al. (2022) found that six daily 3-second maximal eccentric contractions led to a more than 10% increase in muscle thickness within four weeks. Katsura et al. (2019) highlighted that eccentric-focused exercise without equipment (e.g., slow chair sit-downs) performed more than four times a week improved muscle strength and functional fitness more than concentric-focused training over an eight-week period. Additionally, Chen et al. (2017) observed greater gains in muscle strength, balance, and metabolic health markers from twice-weekly descending stair walking sessions over 12 weeks compared to concentric exercise. Eccentric exercise can induce delayed onset muscle soreness (DOMS) when performed for the first time or after a long interval from a previous exercise bout, especially in naïve individuals (Clarkson and Hubal 2002; Proske and Morgan 2001). However, lower-intensity protocols (Sato et al. 2022; Yoshida et al. 2022; Katsura et al. 2019; Chen et al. 2017) have been shown to induce little or no DOMS, and soreness can be minimized by gradually increasing the intensity and volume.

Therefore, the purpose of this study was to investigate the effectiveness of a time-efficient, home-based eccentric-focused resistance exercise intervention in promoting physical activity and improving health-related outcomes in previously inactive individuals. We hypothesized that a short-duration, daily eccentric exercise regimen would lead to significant improvements in key components of physical fitness, including muscle strength, flexibility, and lean body mass, compared to baseline measures. Additionally, we expected that the intervention would be well tolerated by inactive individuals, and the lower perceived effort associated with eccentric exercise would facilitate adherence to the exercise program, contributing to positive changes in both physical and mental health indicators over the intervention period.

## Methods

### Participants

Twenty-four healthy but sedentary individuals (5 men, 19 women) volunteered to participate in the study. Participants were recruited from a convenience sample of staff and community members in and around Edith Cowan University (Perth, Australia), which contributed to the predominance of female participants. Participant activity was assessed using the international physical activity questionnaire (IPAQ) and the total activity time for each participant was converted to MET-minutes per week (Committee 2005). Those who were categorized as inactive or minimally active, without neuromuscular or musculoskeletal disorders, injuries, or other medical conditions that could prevent safe exercise participation, and were not on medication that could influence exercise capacity, were accepted into this study. This study was approved by the Edith Cowan University Human Research Ethics Committee (approval number: 2021–03011-KIRK) and conformed to the Declaration of Helsinki. Participants gave their written informed consent and confirmed that they were physically capable of participating in the study, including the testing procedures.

Twenty-four participants commenced the study, of which two (1 male, 1 female) withdrew due to work and personal reasons unrelated to the study. Thus, the final sample size was 22. The sample size was based on the study by Katsura et al. (2019), which reported large effect sizes (>0.8) for changes in outcome measures similar to those of the present study after 8 weeks of bodyweight eccentric exercise training. The final sample size (n=22) was considered to be adequate to detect possible changes in the outcome measures in the present study. Participants were 50 ± 10 (mean ± SD, range: 32–69) years old, with a height of 174.9 ± 1.8 (172.5–176.5) cm and body mass of 82.7 ± 9.0 (69.9–90.4) kg for men and 165.8 ± 5.9 (157–182) cm and 76.5 ± 13.2 (50.9–104.5) kg for women, respectively.

### Study design

Participants were familiarized with all testing procedures prior to study commencement in a single session which included demonstration and practice of the physical fitness tests described below. Upon study commencement, participants completed a 2-week control period followed by a 4-week daily exercise intervention period. The 2-week control period was used to mitigate a possible learning effect on outcome measures that is regularly observed in physical fitness tests (Hopkins 2000). Outcome measures described below were taken at three time points, before (PRE-1) and after the control period (PRE-2) as well as after the 4-week intervention (POST) between 1 and 2 days after the last training session. At each testing session, participants were asked to arrive fasted for blood tests and body composition measurement.

To monitor daily activity, each participant was provided with a Charge 5 FitBit watch (Fitbit, San Francisco CA, USA) to wear for the study’s duration. Each participant was given a unique email address, which was used by the investigator to track daily steps. Participants were instructed not to change their physical activity other than performing the exercise program provided. They were also asked not to change their diet during the experimental period.

### Exercise program

Following the 2-week control period, participants completed a low-intensity exercise program (Table 2), adapted from the study by Katsura et al. (2019), in which the eccentric phase of each movement was emphasized by modulating movement velocity. Each day, participants completed one set of chair squat, chair recline, wall push-up, and heel drop exercises for 10 repetitions each using a 5-s eccentric (lowering) phase with 1-s concentric (raising) phase. Participants were allowed to choose the time at which they completed the exercises, and the exercises could be performed together or spread throughout the day. Once 10 repetitions could be easily completed for two consecutive exercise sessions (i.e., the exercise provided an RPE score: < 5/10), participants were advised to progress to a more difficult version of the exercise, which then replaced the previous exercise. Several versions of each exercise were provided to participants that progressed in difficulty (Table 2).

Before program commencement, participants were familiarized with the four initial exercises as well as their progressions and a sheet describing the exercises was provided (the program exercises can be found in Online Resource 1). Participants also performed the first exercise session under supervision to ensure that correct technique was used. A password-protected online spreadsheet was used by the participants to log their exercise sessions. This spreadsheet could be accessed by the lead investigator, who monitored exercise adherence throughout the study. All participants were followed up via email after the first week of training to check for any issues or uncertainties surrounding the exercise program.

### Body composition, heart rate, and blood pressure

Participants had their height and body mass recorded using a stadiometer and a calibrated scale, respectively. A whole-body dual-energy absorptiometry (DEXA, Hologic Discovery X, USA) scan was then performed to assess body composition (e.g., lean body mass (LBM), fat mass, and relative body fat percentage). Participants were asked to lay supine on the center of the bed ensuring all body parts were inside the scanning zone. Upon scan completion, participants had their heart rate (HR), systolic blood pressure (SBP), and diastolic blood pressure (DBP) measured by automatic sphygmomanometer (HEM-7122; Omron Healthcare Co., Ltd, Japan).

### Physical fitness tests

#### Isometric mid-thigh pull (IMTP)

The IMTP was used to assess maximal lower body strength (Comfort et al. 2019). Each participant was asked to stand on a set of Pasport force plates (Pasco, Roseville, USA) (one under each foot) attached to a portable IMTP rig (Vald, Newstead, Australia). To assume the correct pull position, each participant was instructed to stand close to the bar so that the thighs made contact, bend the knees while keeping an upright torso, and to grip the bar (with the aid of lifting straps). Minor adjustments were made to the rack height and participant position so that the bar was between the mid-point of the thigh and the iliac crest with the hips and knees slightly bent (125º–145º and 120º–150º, respectively) and the torso upright (Haff et al. 2015; Beckham et al. 2018) to ensure the strongest force output was produced. A warm-up consisting of 3-s pulls at 50%, 75%, then 90% of perceived maximum effort was performed, in which participants were instructed to “stand up” while maintaining grip of the bar (thus producing torque at both the knee and hip). Attempts were separated by 1 min of rest. After the warm-up, participants completed 3 maximal attempts, with a 1-min rest between efforts. The average of the three attempts was used for analysis (Dos' Santos et al. 2017; Comfort et al. 2019).

#### Squat (SJ) and countermovement jump (CMJ)

Each participant was instructed to place their hands on their hips with feet hip-width apart while standing on a force platform (Pasport Force Platform PS-2141; Pasco scientific, CA, USA). For the SJ, participants were instructed to crouch down to a self-selected squat depth (Petronijevic et al. 2018). Once in position, a countdown of “3, 2, 1 jump” was given and on “jump,” participants jumped upwards as high as possible and landed back onto the force platform (Kotani et al. 2022). A 3-s hold of the bottom position was used to eliminate any countermovement. If a countermovement was visually observed by the researcher, the repetition was repeated. For the CMJ, participants were instructed to freely flex the hip, knee, and ankle joints while keeping the hands on the hips. Participants were given three maximal attempts in each jump and the best score was used for analysis. The rest time between jumps was 20 s.

#### Handgrip strength (HG) test

Each participant was asked to stand and hold a hand grip dynamometer (Jamar Smart Hand Dynamometer; Patterson Medical, IL, USA) with the shoulder adducted and neutrally rotated, elbow extended at ~180° and the wrist between 0° and 30° extension and between 0° and 15° ulnar deviation (Innes 1999). Once in position, the participant was asked to apply maximal force for 3 s and the maximal force measured in kg was recorded (Innes 1999). Three trials for each hand were completed, alternating hands and allowing 30 s between attempts. The best of the three attempts for each hand was used for further analysis.

#### Push-up endurance test

Participants were given 2 min to perform as many push-ups as possible. For men, push-ups were conducted on the hands and feet and for women, push-ups were completed on the hands and knees. Participants were instructed to maintain a ~180º hip angle, with hands spaced shoulder width apart and aligned with the participants eyes. In each repetition, the body was lowered so that a 90° angle formed between the upper and the lower arm at the elbow and with the hands aligned with the chest before returning to the starting position. Participants were reminded to maintain a flat back and to avoid flexion or extension at the lumber spine throughout the test. Where correct technique was not adhered to, the repetition was not counted as valid. The test was terminated if any body part other than the feet (or knees) and hands contacted the ground, if the technique faltered following a warning, or upon the participant’s own volition. The number of push-ups successfully completed at test end was recorded and used for analysis.

#### Sit-up endurance test

Participants were instructed to assume a supine position with the knees bent to 90°. During each repetition, the participants hands ran along the top of the upper leg until the fingertips reached the kneecap, before returning to the start position. Participants were instructed to avoid pulling on their clothes to pull the torso upward and to avoid sudden movement of the neck, shoulder, and hips to generate momentum. One repetition was completed every 3 s to a cadence. The test was terminated if the participant fell behind the cadence for 3 consecutive repetitions, or alternatively if they completed the maximum of 100 repetitions. The number of sit-ups successfully completed at test end was recorded and used for analysis.

#### 3-minute step-up test

Each participant put on a heart rate monitor (H10, Polar Electro, Finland) and rested for 5 min in a seated position. During the final minute, heart rate (HR) was averaged and used as the resting value (HR_pre_) for the 3-min step test. Following the HR measurement, the participant was asked to step up and down on a box of adjustable height to a metronome (24 steps/min) for 3 min. Step height was adjusted using the equations *H*_*f*_ = 0·189*I*_*h*_ for females and *H*_*f*_ = 0·192*I*_*h*_ for males, where *H*_*f*_ is the step height and *I*_*h*_ is the height of the participant (Culpepper and Francis 1987). At the end of each minute of the test, HR and rating of perceived effort (RPE; 1–10 scale; displayed in Appendix I) were recorded. Upon test completion, the participant was asked to sit down and then remain seated for 1 min, during which time HR and RPE (1–10 scale) were recorded 5 s and 1 min after sitting (HR_post_, HR_post,1-min_). The change in HR (HR_pre_ to HR_post_) over the 3-min step-up test was used as an indicator of cardiovascular fitness. A recovery score was also calculated using the change in HR from HR_pre_ to HR_post,1-min_. Additionally, RPE was used to assess perceived exertion experienced from the 3-min step test.

#### Sit-and-reach (S&R) flexibility test

Each participant was asked to sit on the floor with legs together, knees extended, and with shoes removed, the soles of the feet placed against the edge of a sit-and-reach box (Figure Finder Flex Tester, Novel Products Inc, USA). The feet were located at 22.9 cm on the scale for all attempts. With arms outstretched, the participant reached forward while sliding their hands along the measuring scale as far as possible without bending the knees (Hartman and Looney 2003). To ensure accurate scoring the stretch was held at maximal range of motion for 2 s before the score was recorded to the nearest 0.5 cm (Hartman and Looney 2003). The best score from three trials was used for analysis.

### Blood sampling and analyses

Participants reported to the laboratory in the morning after 10–12 hours fasting. Blood samples were collected from the antecubital vein using four separate vacutainers: plasma preparation (PPT), serum separating (SST), ethylenediaminetetraacetic acid (EDTA), and fluoride/oxalate tubes (BD Biosciences, Franklin Lakes, New Jersey, USA) (Candlish 2023). After collection, the EDTA tube was labeled, refrigerated, and shipped for HbA1c analysis. The remaining PPT, SST, and fluoride/oxalate tubes were placed at room temperature for 10 min to allow clotting to occur before being centrifuged (Heraeus Multifuge 3 SR, Thermo Fisher Scientific, USA) at 3000 g for 10 min, and aliquoting the supernatant into microtubes for storage at −80 °C before subsequent analysis.

Fluoride/oxalate samples were used for determination of glucose concentrations, while SST samples were used to measure insulin, fructosamine, triglyceride, total cholesterol, low-density lipoprotein cholesterol (LDL), high-density lipoprotein cholesterol (HDL), high-sensitivity C-reactive protein (hsCRP), and HbA1c was assessed using EDTA. Procollagen type 1 N-terminal propeptide (P1NP) and C-terminal telopeptide of type I collagen (CTX-1) were measured to assess bone metabolism using SST and PPT, respectively. Plasma glucose and serum insulin HDL, LDL triglyceride, total cholesterol, and hsCRP were analyzed using the Alinity ci-series clinical chemistry and immunoassay integrated analyzer (Abbott Laboratories, Chicago, Illinois, USA) using a combination of photometric, potentiometric, and biotin interference free-chemiluminescent detection technologies. Fructosamine and HbA1c analyses were performed using the Roche Cobas c501 and Cobas c513 chemistry analyzers, respectively (Roche Diagnostics, Basel, Switzerland). Briefly, fructosamine is a colorimetric assay and HbA1c is a turbidimetric inhibition immunoassay standardized and traceable to the IFCC (International Federation of Clinical Chemistry and Laboratory Medicine) reference method free from interference with most known HbA1c variants. Laboratory analyses were conducted by PathWest Laboratory Medicine WA in Perth, Western Australia, according to standard accredited clinical laboratory procedures.

### Questionnaires

Participants were asked to complete a 36-item short form survey (SF-36) and 6-point subjective vitality scale (SVS) to assess physical and mental wellbeing (McHorney et al. 1993). SF-36 physical and mental health scores were calculated using the RAND scoring instructions (RAND n.d.). The sum of the 6-point vitality scale for six questions was calculated with a higher score indicating a better condition.

At the end of the study, participants completed an exit survey which asked how they felt (stronger, fitter and healthier) after the 4-week exercise intervention as well as whether they enjoyed the program. For each question, a five-point Likert scale was used (1 = strongly disagree, 2 = disagree, 3 = neither agree nor disagree, 4 = agree, 5 = strongly agree). The mean of these responses was then calculated and reported. Participants were also asked whether they planned to exercise in future using the following scoring: 1 = yes, using the same eccentric-biased regime, 2 = yes, but doing my own thing, 3 = no, and 4 = unsure. Finally, participants were asked “how likely are you to recommend this exercise program to a friend or family member”? Scoring for this question ranged between 1 and 10, with 10 being the strongest recommendation score. At 4 weeks post-intervention, participants were asked whether they were currently exercising, and if so, what type of exercise they were performing.

### Statistical analyses

Intraclass correlation coefficient (ICC) estimates and their 95% confident intervals were calculated based on a mean-rating (k = 2), absolute-agreement, and one-way mixed-effects model using the two measures in the control period to establish the test–retest reliability of the outcome measures. ICC estimates were interpreted as poor (<0.5), moderate (0.5 and 0.75), good (0.75 and 0.9), and excellent (>0.90) (Koo and Li 2016).

The data were assessed for assumptions of normality by a Shapiro–Wilk test and for sphericity by a Mauchly’s sphericity test. In instances where normality was violated, a Friedman test was used. A one-way repeated-measures analysis of variance was used to test for changes in the dependent variables over the three time points. In the case of a significant time effect, a Holm’s sequential Bonferroni correction was performed to compare the values between time points (Holm 1979). Eta squared values (*η*^*2*^) were also reported as a measure of factor variation size, which were interpreted as small (*η*^*2*^ = 0.01), medium (*η*^*2*^ = 0.06), and large (*η*^*2*^ = 0.14) effect (Lakens 2013). The significance level was set to *p* ≤ 0.05.

Additionally, the changes from PRE-1 to PRE-2 (control period) were compared to the changes from PRE-2 to POST (training period) using a linear mixed-effects model (LMM). The LMM was specified with the change scores as the dependent variable, the period (control vs. intervention) as a fixed effect, and subject as a random effect to account for the repeated-measures design. This model allowed for the assessment of the intervention effect while controlling for individual variability and potential confounders. This comparison aimed to ensure that any significant changes observed during the intervention period were not present during the control period, thereby supporting the causal effect of the training program. To reject the null hypothesis, the following conditions had to be simultaneously met: 1) ICC in the control period was expected to exceed 0.70, indicating moderate reliability, 2) the one-way repeated-measures ANOVA had to detect a statistically significant effect of time, demonstrating that there were differences between the time points, 3) post hoc comparisons with Holm’s sequential Bonferroni correction had to identify significant differences between PRE-2 vs. POST, and 4) the LMM had to reveal a significant fixed effect of time (control vs. intervention) on the change scores, confirming that the changes observed during the intervention period were significantly different from those observed during the control period.

To explore whether age or body mass influenced the training outcomes, an additional LMM analysis was performed with time point as a fixed factor and age and body mass as covariates. Interaction terms (time point × age and time point × body mass) were also included to determine whether changes in outcome measures differed across individuals with varying age or body mass. This analysis was applied to all performance-based measures where significant time effects were detected.

A within-subject correlation analysis was performed to quantify the relationships between variables across time points. This analysis utilized the sum of squares and residual sum of squares outputs computed by the analysis of covariance (ANCOVA), as recommended by Bland and Altman (1995). By incorporating data collected from each participant across multiple time points into a single correlation, this method allowed for the computation of an overall correlation between variables over time. The strength of relationship was quantified as trivial (*r* < 0.1), small (*r* = 0.10–0.29), moderate (*r* = 0.3–0.49), large (*r* = 0.5–0.69), very large (*r* = 0.7–0.89), and nearly perfect (*r* ≥ 0.9) (Cohen 2013). All statistical testing was performed using Jamovi version 2.3.21 (Jamovi project, 2018). Data are presented as mean ± standard deviation (SD).

## Results

### Test–retest reliability

ICCs demonstrated good (0.75 and 0.9) to excellent (>0.90) reliability for most measures. However, SVS and HR_pre_ to HR_post,1-min_ exhibited moderate (0.6 and 0.65, respectively) reliability. Table 1 shows specific ICC values.

**Table 1:** Test–retest reliability assessed by intraclass correlation coefficient (ICC) and 95% confidence interval of outcome measures

	ICC	95% Confidence Interval	CV (%)
Resting heart rate	0.72	(0.45–0.87)	8
Systolic blood pressure	0.89	(0.76–0.95)	5
Diastolic blood pressure	0.93	(0.85–0.97)	4
Body mass	1	(1–1)	1
Lean body mass	0.99	(0.98–1)	1
Fat mass	1	(0.99–1)	2
Isometric mid-thigh pull	0.92	(0.83–0.97)	7
Grip strength left	0.9	(0.77–0.96)	5
Grip strength right	0.91	(0.80–0.96)	6
Squat jump	0.97	(0.93–0.99)	7
Countermovement jump	0.98	(0.94–0.99)	6
Push-up	0.92	(0.82–0.97)	14
Sit-up	0.82	(0.60–0.92)	16
Sit & reach	0.97	(0.93–0.99)	6
3-min step test HR_pre_	0.86	(0.69–0.94)	6
3-min step test HR_post_	0.95	(0.87–0.98)	3
3-min step test ∆ HR_pre_ - HR_post_	0.87	(0.70–0.94)	6
3-min step test ∆ HR_pre_ - HR_post,1-min_	0.65	(0.32–0.84)	51
SF-36 physical	0.72	(0.42–0.88)	10
SF-36 mental	0.75	(0.47–0.89)	18
Subjective vitality scale	0.6	(0.25–0.82)	16
Glucose	0.8	(0.57–0.92)	6
Insulin	0.73	(0.45–0.88)	27
HbA1c	0.89	(0.76–0.96)	5
Fructosamine	0.67	(0.34–0.85)	9
Triglyceride	0.79	(0.56–0.91)	26
Total cholesterol	0.73	(0.45–0.88)	13
LDL	0.77	(0.51–0.90)	13
HDL	0.72	(0.43–0.88)	14
hsCRP	0.75	(0.48–0.89)	39
P1NP	0.94	(0.86–0.98)	11
CTX-1	0.91	(0.80–0.96)	16

### Program adherence

Exercise program adherence was 91 ± 12% (range: 18–28 sessions out of 28 sessions). Over the 4-week intervention, 14 participants progressed from the chair squat to the one-leg biased chair squat and two participants progressed to the pistol squat. In the push exercise, four participants progressed to the one-arm push-up, two progressed to the table push-up, nine participants progressed to the knee push-up, and three participants (men) progressed to the push-up. In the abdominal exercise, seven participants progressed to the leg straight chair recline back and seven progressed to the sit-up. In the calf exercise, seven participants progressed to the heel drop overstretch and seven progressed to the 1-leg heel drop overstretch exercise (see Table 2 for exercise program summary). Daily steps did not differ between control (7437 ± 1856 steps per day) and exercise (7396 ± 1799 steps per day) periods (*p* = 0.966).

**Table 2:** Summary of exercise adherence and progression for participants in different exercise categories over the 4-week intervention

Exercise category	Progression	Exercise	Participants completed (n)	Sessions completed	RPE	Participants finished at exercise (n)
Legs	1	Chair Squat	21	14 ± 10 [1–28]	5.7 ± 1.7 [3.0–9.7]	6
	2	One-leg chair squat	16	17 ± 8 [1–28]	5.9 ± 1.6 [3.0–7.8]	14
	3	Pistol squat	2	5 ± 4 [2–7]	7.1 ± 0.2 [7.0–7.3]	2
Chest	1	Wall push-up	22	12 ± 9 [1–29]	5.6 ± 1.8 [2.0–9.2]	4
	2	One-arm wall push-up	8	9 ± 6 [1–18]	7.7 ± 1.8 [5.5–10]	4
	3	Table push-up	3	16 ± 12 [4–27]	6.0 ± 2.6 [3.0–9.8]	2
	4	Knee push-up	10	18 ± 8 [1–25]	7.1 ± 1.7 [3.0–9.8]	9
	5	Push-up	3	14 ± 12 [6–28]	5.8 ± 3.9 [3.0–8.6]	3
Abdominals	1	Chair recline back	21	15 ± 10 [1–29]	6.3 ± 1.6 [3.0–9.7]	8
	2	Chair Recline Back (legs straight)	12	13 ± 8 [1–27]	6.0 ± 1.7 [3.0–8.5]	7
	3	Sit-up	7	14 ± 8 [2–24]	7.0 ± 2.0 [3.0–9.0]	7
Calves	1	Heel drop	9	11 ± 9 [4–28]	6.0 ± 1.3 [4.0–7.5]	1
	2	Heel drop overstretch	21	20 ± 7 [4–28]	5.9 ± 1.9 [2.1–10.0]	18
	3	1-leg heel drop overstretch	3	9 ± 10 [1–20]	6.5 ± 0.5 [6.1–7.0]	3

“Participants completed (n)” indicates the number of participants who performed each exercise during the intervention. “Participants finished at exercise (n)” represents the number of participants for whom the listed exercise was the most challenging level achieved. Exercises are listed in order of increasing difficulty within each category. Data are presented as mean ± SD (range)

*RPE* Rate of perceived exertion of each exercise

### General health and body composition

As shown in Table 3, no significant changes in body mass, fat mass, lean mass, resting heart rate, or systolic or diastolic blood pressure were detected between PRE-1 to PRE-2 or PRE-2 to POST. Moderate within-subject correlations across time points were observed in LBM and push-ups (*r* = 0.41, *p* = 0.007) and LBM and sit-ups (*r* = 0.39,* p* = 0.057).

**Table 3:** Mean ± SD (range) body mass, fat mass, % fat, bone mineral content, lean mass, resting heart rate (HR), systolic (SBP) and diastolic blood pressure (DSB), SF-36, and subjective vitality scale (SVS) scores at baseline (PRE-1), after 2-weeks of control period (PRE-2), and after 4-weeks of the exercise intervention period (POST)

	PRE-1	PRE-2	POST
Body mass (kg)	77.7 ± 12.6 (50.9–104.5)	77.9 ± 12.7 (51.8–105.2)	78.0 ± 13.0 (51.1–105.6)
Fat mass (kg)	29.1 ± 9.2 (12.6–48.2)	29.2 ± 9.1 (13.3–46.0)	29.1 ± 9.5 (13.5–49.0)
Fat (%)	36.4 ± 7.2 (24.6–46)	36.5 ± 7.3 (24–46.3)	36.3 ± 7.5 (24.1–46.4)
Bone mineral content (kg)	2.5 ± 0.3 (2.0–3.3)	2.4 ± 0.3 (2.0–3.4)	2.5 ± 0.3 (1.9–3.3)
Lean mass (kg)	46.8 ± 6.4 (36.5–60.4)	46.8 ± 6.7 (37.2–61.4)	47.2 ± 6.9 (36.0–63.0)
Heart rate (bpm)	64.1 ± 7.4 (53 –81)	62.9 ± 10.6 (46–95)	63.6 ± 8.6 (48–86)
Systolic blood pressure (mmHg)	125.5 ± 19.7 (99–178)	124.6 ± 19.0 (100–184)	120.7 ± 14.6 (97–148)
Diastolic blood pressure (mmHg)	74.6 ± 10.6 (59–97)	73.9 ± 11.5 (53–99)	71.5 ± 10.4^*^ (53–97)
SF-36 physical health	47.8 ± 8.3 (31.4–62.4)	50.0 ± 10.6 (26.1–65.2)	59.2 ± 9.6 (22.1–58.9)

∗Significant (p < 0.05) difference from PRE-1 and/or PRE-2

### Physical fitness

**Fig. 1:**
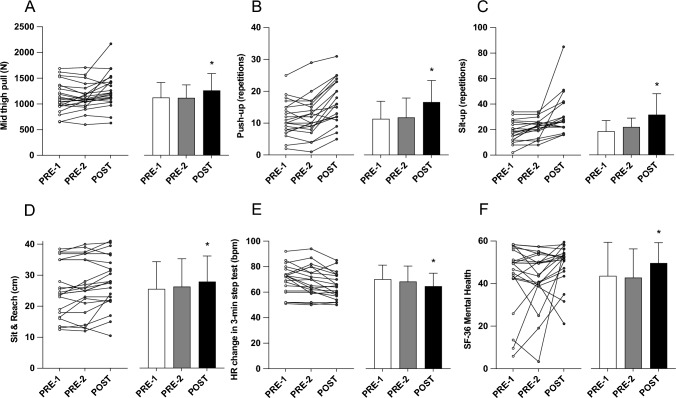
Individual (left) and mean ± SD (right) isometric mid-thigh pull force (**A**), push-up repetitions (**B**), sit-up repetitions (**C**), sit & reach distance (**D**), heart rate change in 3-min step test (**E**), and SF-36 mental health score (**F**) scores. ∗significant (p < 0.05) difference from PRE-1 and/or PRE-2

No significant changes in grip strength (left or right hands), SJ, or CMJ height were detected over time (Table 4). No changes were observed over the control period (PRE-1 to PRE-2) for IMTP, push-ups, or S&R (*p* > 0.05). After training IMTP increased from 1132.6 ± 252.6 N at PRE-2 to 1275.2 ± 334.5 N at POST (*p* = 0.012, *η*^*2*^ = 0.05). The change in IMTP was greater over the training (PRE-2 to POST) period (142.6 ± 722.7 N) compared to the control period (− 6.4 ± 109.0 N, *p* = 0.005). Neither age (*p* = 0.224) nor body mass (*p* = 0.641) significantly influenced the changes in IMTP. Push-up performance improved from 11.8 ± 6.2 repetitions at PRE-2 to 16.6 ± 6.8 repetitions at POST (*p* < 0.001, *η*^*2*^ = 0.134). The change in push-up performance was greater over the training period (4.6 ± 3.3 repetitions) compared to the control period (0.4 ± 2.3 repetitions, *p* = 0.001). Age significantly influenced push-up performance (*β* = −0.246, *p* = 0.037), but no interaction effect was detected (*p* = 0.164). Sit & reach (S&R) distance also increased from 26.3 ± 9.0 cm at PRE-2–28 ± 8.3 cm at POST (*p* = 0.039, *η*^*2*^ = 0.014); however, the change in S&R distance between the control period (0.8 ± 2.2 cm) and the training period (1.6 ± 3.0 cm, *p* = 0.273) did not differ significantly. The change in S&R was not affected by age (*p* = 0.594) or body mass (*p* = 0.052). A detailed visual summary of these results is shown in Fig. 1.

**Table 4:** Mean ± SD (range) left- and right-hand grip strength, and squat and countermovement jump heights at baseline (PRE-1), after the 2-week control period (PRE-2), and after the 4-week exercise intervention period (POST)

	PRE-1	PRE-2	POST
Grip strength-left (kg)	32.1 ± 6.0 (22.5–46.6)	31.1 ± 5.1 (19.7–43.4)	33.0 ± 7.4 (21.3–52.2)
Grip strength-right (kg)	34.8 ± 6.8 (20.4–49.5)	34.0 ± 6.6 (20.7–47.1)	35.4 ± 7.9 (21.1–55.2)
Squat jump (cm)	14.6 ± 5.5 (5.0–30.1)	14.7 ± 6.0 (4.7–34.2)	15.3 ± 6.1 (6.2–31.2)
Countermovement jump (cm)	15.5 ± 6.0 (6.8–32.8)	15.6 ± 6.4 (5.2–33.9)	16.1 ± 6.3 (6.5–32.8)

No significant differences were observed within measures

The number of sit-ups performed increased from 17.6 ± 9.2 repetitions at PRE-1 to 20.8 ± 8.4 repetitions at PRE-2 (p = 0.029), indicating the presence of an order or learning effect. Sit-ups increased further to 31.3 ± 16.2 repetitions at POST (*p* < 0.001, *η*^*2*^ = 0.206; Fig. 1). The change in sit-up performance was greater over the training period (9.5 ± 14.2 repetitions) than the control period (2.9 ± 4.4 repetitions, p = 0.048). The good reliability of the sit-up test (ICC = 0.85, 95% CI: 0.60–0.92) also supports greater improvements at post-training compared to the control period. Neither age (*p* = 0.331) nor body mass (*p* = 0.659) significantly influenced sit-up performance.

The HR change (HR_pre_ to HR_post_) in the 3-min step test decreased from 68.6 ± 12.1 bpm at PRE-2 to 65 ± 10.2 bpm (*p* = 0.015, *η*^2^ = 0.041) at POST, with no change observed over the control period (Fig. 1). However, there was no significant difference observed between the control (− 1.6 ± 5.6 bpm) and training (− 3.3 ± 5.3 bpm, p = 0.302) periods. HR change was affected by neither age (*p* = 0.578) nor body mass (p = 0.99). RPE after the 3-min step test decreased from 6.1 ± 1.7 at PRE-2 to 5.4 ± 1.4 at POST (*p* < 0.001, *η*^*2*^ = 0.046), with no change observed in the control period. However, no between-condition difference was observed (*p* = 0.063). Again, no significant effect of age (*p* = 0.97) or body mass (*p* = 0.896) on RPE was observed. No change in HR recovery from HR_pre_ to HR_post,1-min_ was observed over the control period or with training.

### Blood markers

Throughout the study, HbA1c exceeded the reference limit in two participants, while LDL, HDL, hsCRP, insulin, and glucose exceeded the limit in three, five, four, one, and two participants, respectively, across all time points. Notably, some participants who were initially outside the reference range during the control period returned within the references range at POST; this included one participant for cholesterol, LDL, HDL, triglycerides, and two participants for hsCRP. Overall, there were no significant changes in blood markers observed over time (see Table 5).

**Table 5:** Mean ± SD (range) plasma glucose, insulin, HbA1c, triglyceride, total cholesterol, low-density lipoprotein cholesterol (LDL), high–density lipoprotein cholesterol (HDL), high-sensitivity C-reactive protein (hsCRP), procollagen type 1 N-terminal propeptide (P1NP), and C-terminal telopeptide of type I collagen (CTX-1) concentrations at baseline (PRE-1), after the 2-week control period (PRE-2), and after the 4-week exercise intervention period (POST)

	PRE-1	PRE-2	POST	Reference Interval
Glucose (mmol/L)	5.0 ± 0.7 (4.2–6.9)	4.9 ± 0.7 (4.0–6.6)	4.8 ± 0.8 (3.9–7.2)	3.0–5.4
Insulin (mU/mL)	5.7 ± 3.2 (1.7–15.3)	6.3 ± 3.1 (2.3–13.5)	5.7 ± 3.2 (1.8–16.3)	<12
HbA1c (mmol/mol)	34.7 ± 6.0 (25.0–50.0)	36.0 ± 6.4 (29.0–55.0)	35.7 ± 6.0 (29.0–55.0)	<42
Fructosamine (umol/L)	210.9 ± 38.8 (159.3–307.5)	214.4 ± 28.2 (170–280.3)	204.5 ± 29.3 (136.4–258.9)	205–285
Triglyceride (mmol/L)	0.86 ± 0.44 (0.39–2.03)	0.89 ± 0.53 (0.37–2.46)	0.86 ± 0.35 (0.34–1.65)	<1.7
Total cholesterol (mmol/L)	4.01 ± 0.96 (2.34–5.53)	4.13 ± 0.92 (2.47–5.71)	3.90 ± 0.88 (1.68–5.42)	<5.5
LDL (mmol/L)	2.57 ± 0.72 (1.14–3.80)	2.69 ± 0.72 (1.20–4.00)	2.52 ± 0.69 (0.84–3.79)	<3.0
HDL (mmol/L)	1.14 ± 0.29 (0.58–1.66)	1.16 ± 0.30 (0.62–1.7)	1.11 ± 0.29 (0.43–1.58)	>1.0
hsCRP (mg/L)	0.99 ± 0.88 (0.14–3.72)	0.98 ± 0.61 (0.09–2.08)	0.92 ± 0.64 (0.05–2.59)	<1.0
P1NP (ug/L)	51.5 ± 23.6 (15.1–110.0)	47.9 ± 22.4 (16.6–114.0)	47.2 ± 20.9 (15.7–101.0)	M: 15–80, F: 15–90
CTX–1 (ng/L)	411.5 ± 224.6 (115.0–923.0)	398.9 ± 198.6 (113.0–827.0)	397.1 ± 229.1 (86.0–555.0)	M: 100 – 600 F: 150–800 (Menopausal: 50–800)

Reference interval is provided for each measure. No significant differences were observed within measures

### Questionnaires

No significant changes were observed in the SF-36 physical health (*p* = 0.316) (Table 3) or mental health components over the control period. A significant increase in mental health from 42.9 ± 13.5 to 49.8 ± 9.6 (*p* = < 0.001, *η*^*2*^ = 0.218) was observed after training (Fig. 1). However, there was no significant difference between the control (− 4.8 ± 18.5) and training (10.8 ± 17.5, *p* = 0.366) periods. Neither age (*p* = 0.446) nor body mass (*p* = 0.236) significantly influenced SF-36 mental health. Moderate within-subject correlations across time points were observed for mental health and push-ups (*r* = 0.34, *p* = 0.026) and mental health and HR_pre_ to HR_post_ in the 3-min step test (*r* = 0.34, *p* = 0.028).

The subjective vitality score (SVS) decreased from 28.7 ± 7.8 at PRE-1 to 24.9 ± 7.0 at PRE-2 (*p *= 0.042) and then increased to 29.9 ± 6.7 at POST (*p* < 0.042, *η*^*2*^ = 0.075). The change in SVS was greater over the training period (6.1 ± 10.4) than the control period (− 5.0 ± 9.0, *p* = 0.048). No significant effect of age (*p* = 0.894) or body mass (*p* = 0.853) on SVS was evident. The ICC for SVS in the control period was 0.6 (95% CI 0.25–0.82), indicating moderate reliability but falling below the predefined threshold. A moderate within-subject correlation across time points was observed for SVS and S+R (*r* = 0.35,* p* = 0.02).

Participants reported that they felt “stronger” (81 ± 10%), “fitter” (71 ± 16%), and “healthier” (78 ± 12%) after the 4-week exercise intervention. “Enjoyment” of the program was high (89 ± 10%). When asked about future exercise, 64% of participants said they would continue the exercise protocol, 32% would exercise in a different way, 5% were unsure, and no participant indicated that they would cease exercising. Recommendation of the exercise program to family or friends was 91 ± 10%. At 4 weeks post-intervention, 10 participants had commenced a new exercise intervention and were therefore excluded from the follow-up analysis. Of the 12 participants eligible to be surveyed, 10 (83%) reported continuing to exercise, either using the same exercises or incorporating traditional resistance, cardiovascular, or other exercise methods (e.g., yoga).

## Discussion

This study examined whether a daily 5-min bodyweight eccentric-focused program could improve markers of physical fitness, health, body composition, and mental wellbeing in sedentary individuals over four weeks, as well as the program’s impact on exercise behaviors after the intervention period. The results revealed improvements in muscle strength, flexibility, strength endurance, and mental health following the exercise program, suggesting that even a small amount of daily exercise can provide substantial and detectible benefits in sedentary individuals. Additionally, the program effectively encouraged sedentary individuals to incorporate exercise into their daily routine.

Regarding program adherence, the participants adhered to the exercise program well over the 4-week intervention period (89%), with almost all sessions being completed and tracked within the spreadsheet. Participants reported that both the short exercise duration and time flexibility were facilitators of adherence. These elements should therefore be considered when designing future exercise programs, particularly for individuals who have limited time availability for exercise. At the conclusion of the exercise program, participants noted “feeling” stronger, fitter, and healthier. Furthermore, all participants showed a willingness to continue some form of exercise following the exercise program, and at 4-week post-intervention, most participants reported they had continued performing the minimal-dose program or had commenced regular resistance, cardiovascular, yoga exercise, or a combination of these activities. These results support the idea that, in addition to a reduced perceived time burden, an individual’s perception of their own competence can significantly influence their adherence to exercise programs (Whaley and Schrider 2005). Further work is warranted is this area; however, the current study suggests that a “minimalistic” exercise protocol might be a solution to combat sedentary behavior by producing detectible outcomes in minimal time and thus increasing future active behavior.

The mental health component of the SF-36 significantly improved following the exercise intervention. This result aligns with previous research that regular physical activity can improve mental health and lessen symptoms of depression, anxiety, and stress (DiLorenzo et al. 1999; Peluso and De Andrade 2005). Given the complexity of mental health, it is possible that factors such as social interaction, placebo effects, or participants’ pre-existing mental health conditions may have contributed to these improvements. However, the results of the present study suggest that a few weeks of 5-min daily bodyweight eccentric-focused exercise can be effective at improving mental wellbeing. Additionally, while subjective vitality slightly decreased from PRE-1 to PRE-2, it increased significantly over the 4-week training period. The decline at PRE-2 could be attributed to the moderate reliability (ICC: 0.6, 95% CI 0.25–0.82) observed within the measurement, obscuring true effects. Alternatively, the decrease over the control period might have resulted from anticipatory uncertainty (Greco and Roger 2001), where participants knew they would commence the exercise intervention after the testing session (which was unfamiliar to the non-exercising participants), or that participants were excited to commence and finish the study which inflated the PRE-1 and POST scores, respectively. In addition to the observed improvements in mental health scores following the exercise intervention, moderate correlations were noted between SVS and S&R as well as between the SF-36 and both the push-up score and HR during the 3-minute step test. These findings highlight at least some interconnectedness between physical and mental health outcomes, which is an important outcome of the study and would be of interest to examine further in future.

Over the 2-week control period, all outcome measures exhibited moderate-to-excellent reliability, suggesting that the observed changes following the exercise intervention were likely genuine improvements rather than measurement noise or improvements due to repeated measurements (i.e., learning). Importantly, the changes in IMTP (13%), push-up (66%), sit-up (51%), and S&R (9%) all exceeded the estimated measurement errors (Table 1), providing further evidence for the effectiveness of the 4-week eccentric exercise program.

It has been suggested that the closer the mechanical specificity of a training exercise to a functional outcome, the greater the transfer of performance gain (Appleby et al. 2019), and the results of the present study support this concept. Significant improvements were observed in the push-up and sit-up tests following the exercise program, which were exercises that either directly or very similarly matched the push-up and sit-up training exercises. Also, the IMTP test requires simultaneous hip and knee extension, similar to the chair squat training exercise. In contrast, SJ and CMJ tests, which are often used as analogues for muscle power production, did not improve, which may feasibly be explained by a lack of high velocity movement training and/or a lack of intention to move fast during the training exercises (Kawamori and Newton 2006). Overall, the results highlight that small amounts of exercise can improve physical capacity, but that the exercise may need to be specific to desired tasks.

The S&R test, which provides a general assessment of flexibility at the torso, lower back and posterior upper- and lower-limb muscles (Hartman and Looney 2003), improved by 9% following the 4-week intervention. While this improvement did not meet all statistical criteria for the primary analysis, it aligns with findings from Barbosa et al. (2002), who observed a 13% improvement in S&R distance following 10 weeks of weighted resistance exercise in elderly women. The notable 9% increase in just 4 weeks of 5-min daily exercise in the present study suggests positive trends attributable to the intervention. In our study, we applied stringent criteria to reject the null hypothesis. However, the observed improvement in S&R test scores indicates that the training program may have broader benefits that warrant further investigation. Whether this flexibility increase positively and directly impacted movement capacity is unclear; however, moderate correlations (*r* = 0.59) have been previously reported between sit & reach ability and functional movement screen scores (Lockie et al. 2015). Therefore, it seems possible that the increase in S&R distance may contribute to improvements in activities of daily living. Speculatively, it may also have contributed to the participants’ increased feelings of vitality as observed by the moderate correlation (*r* = 0.35) between changes in SVS and S&R across the study period and the perceptions of feeling fitter and healthier noted in the exit survey.

The HR response in the step test was reduced following the exercise program (Fig. 1). Because the total work (step height and time) was unchanged, this result suggests that cardiorespiratory efficiency improved. This might be due to an improvement in movement economy and/or improved strength capacity, such that the requisite forces were produced but at a lower activation level following the exercise intervention, which should lower heart rate (Lay et al. 2002). In confluence with heart rate, the step test was perceived as being less effortful following the exercise program, supporting the idea that cardiorespiratory efficiency improved.

It should be noted that no significant between-condition differences were observed in the HR and RPE responses to the 3-min step test, suggesting that these changes may not be solely due to the training intervention. However, the reduced perceived effort could still be of practical significance, as individuals are more likely to complete activities if the expected effort is minimal (Cheval and Boisgontier 2021), and such changes might have contributed to the participants’ higher vitality and fitness perceptions after the training. Therefore, it is possible that the 5-min exercise program could develop into a more robust exercise program as an individual’s fitness improves. This concept is supported by the results of the exit survey, in which all participants noted their desire to continue exercising past the 4-week intervention in the exit survey. Future work is needed to confirm this theory.

Blood pressure assessment is an important predictor of future cardiovascular risk (Cornelissen and Smart 2013). In the present study, no change in diastolic blood pressure was observed, although the mean score was 2 mmHg lower after the 4 weeks of training, which is similar to that reported in previous literature where low-intensity dynamic resistance exercise was performed (95% CI − 1.9 to 2.5 mmHg) (Cornelissen and Smart 2013). With respect to systolic blood pressure, a 5 mmHg mean reduction has been shown to reduce the risk of a major cardiovascular event by about 10% (Rahimi et al. 2021). In the present study, systolic blood pressure did not show a significant change, but 7 out of 22 participants showed 5 mmHg or greater reductions following the training, indicating a possible benefit of the exercise program in those individuals. It would be interesting to see whether a clinically relevant reduction is achieved if the intervention duration was extended.

No statistically significant change was observed in lean body mass over time. However, a large variability in lean body mass change was noted, with 13 individuals gaining (1.3 ± 0.6 kg) lean mass and nine losing it (− 1.0 ± 0.5 kg). This variability might be influenced by each participant’s genetic predisposition to lean mass accrual, and the propensity to progress to the more difficult exercises within the program (Erskine et al. 2010; Van Vossel et al. 2023). The average daily steps completed by participants throughout the study were similar between the control and exercise periods, suggesting that a change in general activity (due to exercise awareness) did not likely influence the results. Food intake (i.e., calorie and protein intake) could impact lean mass accrual and these factors, in part, could have influenced the variation in lean mass responses seen between participants (Volek 2004). As such, this should be monitored along with daily physical activity if this protocol is used for real-world applications. A moderate correlation was observed between lean mass and the push-up (*r* = 0.41) and sit-up (*r* = 0.39) tests, indicating that changes in DEXA-derived lean body mass may have had an impact on strength-endurance tasks. However, no other correlations with performance outcomes were observed, suggesting that these changes did not affect maximal strength, flexibility, or movement economy.

No significant changes in any blood measures were observed. This lack of change might be explained by the participants recruited to the study being healthy and most having blood measures within normal ranges at baseline. Thus, the opportunity for improvement was relatively small. However, previous research has shown that resistance exercise can benefit individuals without diagnosed metabolic or cardiovascular diseases. For example, Prabhakaran et al. (1999) observed decreases in total cholesterol (from 4.60 to 4.26 mmol/L) and LDL cholesterol (from 2.99 to 2.57 mmol/L) following 14 weeks of high-intensity (85% 1-RM) resistance training in 24 premenopausal women. Ahmadi and Gharibi (2011) had 30 healthy male participants complete either moderate-intensity resistance training (45–55% 1-RM) or high-intensity resistance training (80–90% 1-RM) for 6 weeks. Both groups experienced reductions in LDL cholesterol (moderate-intensity − 0.35 mmol/L vs. high-intensity − 0.31 mmol/L) and total cholesterol (moderate-intensity − 0.32 mmol/L vs. high-intensity − 0.29 mmol/L), although HDL cholesterol was only increased in the high-intensity group (+ 0.14 mmol/L). As these studies used external resistance loads, it might be that higher intensity exercise is necessary to improve blood cholesterol levels. Additionally, participants completed a longer training program with substantially greater exercise volume (load × sets × reps). As exercise volume has been shown to influence cholesterol values (Aadahl et al. 2007), it is possible that greater training volume is needed to produce changes in cardiovascular health markers.

Some study limitations and delimitations should be considered. Firstly, there was a predominance of female participants and a relatively large age range. This necessitates more data in men to determine if outcomes might be sex specific. However, relative strength improvement following resistance exercise tends to be similar between men and women (Gentil et al. 2016; Tracy et al. 1999). Thus, the relative strength changes observed in the female participants of this study are likely representative of what might occur in men. While it would have been of interest to study the minimal-dose exercise effects in distinct age brackets (such as young, middle-aged, and older individuals) to capture age-specific adaptations, we prioritized the recruitment of participants across the ages 30–69 years to better represent the whole working demographic where sedentary behavior is prevalent. Notably, the analysis revealed that neither age nor body mass significantly influenced the changes in the outcome measures. While older individuals typically started from a lower baseline, their rate of improvement was comparable to that of younger participants, indicating that the exercise program was effective across a broad spectrum of ages. Secondly, no control group was included in this study, although a control period was implemented prior to the intervention. This may introduce systematic biases or learning effects that could influence participants' responses or performances over time. Additionally, it should be noted that the control period was shorter than the intervention period, potentially impacting the stability of measurements and interpretation of the observed changes. To address these concerns, inter-day reliability within the control period was assessed using the one-way mixed-effects intraclass correlation coefficient with absolute agreement, with a predefined threshold for acceptable reliability set at 0.70. A third limitation was the absence of direct training monitoring and supervision throughout the home-based exercise program, which may have contributed to variability in individual responses (see Fig. 1). Additionally, the prescribed 5-second eccentric contractions, chosen to ensure sufficient workload in an unsupervised setting, may have been challenging to time accurately without guidance, potentially contributing to response variability. Despite this, the intervention was still effective for most participants, demonstrating the potential of self-directed exercise routines in real-world settings. The improvement in strength, flexibility, and movement economy observed in the present study highlights the benefits of even a small amount of exercise. In future, the longitudinal benefits of such an exercise routine might be examined in various workplaces to determine whether exercise “snacks” can be used to reduce the development of chronic disease and increase worker attendance and performance across the working lifespan. Additionally, future exercise snack models could explore the efficacy of incorporating progressive overload strategies, including eccentric overload, varying eccentric deceleration rates, or optimizing eccentric durations (e.g., 3 vs. 5 seconds), to enhance execution ease and ensure consistent adaptations over time.

In conclusion, the 5-min, home-based, eccentric-focused exercise program performed for 4 weeks improved muscle strength, flexibility, and movement economy in healthy but sedentary individuals. These adaptations were greater in tests that more closely matched the exercises performed in the training program (e.g. push-up and IMTP but not vertical jump tests), suggesting that programs should be set with consideration of the main movement goals of the individual. Additionally, the program improved mental wellbeing as shown by improvements in SF-36, SVS, and perceptions of health, fitness, and strength. Importantly, participants exhibited strong adherence to the program and continued to exercise beyond the intervention period. The program may therefore serve as a foundation for establishing a consistent exercise routine in individuals currently leading sedentary lifestyles.

## Supplementary Information

Below is the link to the electronic supplementary material.

Supplementary file1 (PDF 2227 KB)

## Data Availability

All data generated or analysed during this study are included in this published article.

## Abbreviations

1-RM: One-repetition maximum
CMJ: Countermovement jump
CTX-1: C-terminal telopeptide of type I collagen
DBP: Diastolic blood pressure
DEXA: Dual-energy X-ray absorptiometry
DPB: Diastolic blood pressure
EDTA: Ethylenediaminetetraacetic acid
HbA1c: Hemoglobin A1c
HDL: High-density lipoprotein
HR: Heart rate
HR_pre_: Pre-exercise heart rate
HR_post_: Post-exercise heart rate
HR_post,1-min_: Heart rate at 1-minute post-exercise
hsCRP: High-sensitivity C-reactive protein
IMTP: Isometric mid-thigh pull
IPAQ: International physical activity questionnaire
LMM: Linear mixed-effects model
LDL: Low-density lipoprotein
P1NP: Procollagen type 1 N-terminal propeptide
RPE: Rating of perceived exertion
SBP: Systolic blood pressure
SF-36: 36-Item short form survey
SJ: Squat jump
SST: Serum separating tubes
SVS: Subjective vitality scale
S&R: Sit-and-reach test
V-Up: An abdominal exercise
WHO: World health organization
wk: Week
